# A New Approach Based on Collective Intelligence to Solve Traveling Salesman Problems

**DOI:** 10.3390/biomimetics9020118

**Published:** 2024-02-16

**Authors:** Mustafa Servet Kiran, Mehmet Beskirli

**Affiliations:** 1 Department of Computer Engineering, Konya Technical University, 42250 Konya, Türkiye; 2 Department of Computer Engineering, Karamanoğlu Mehmetbey University, 70100 Karaman, Türkiye

**Keywords:** ant system algorithm, collective intelligence, footprint mechanism, path construction, path improvement, traveling salesman problem

## Abstract

This paper presents a novel approach based on the ant system algorithm for solving discrete optimization problems. The proposed method is based on path construction, path improvement techniques, and the footprint mechanism. Some information about the optimization problem and collective intelligence is used in order to create solutions in the path construction phase. In the path improvement phase, neighborhood operations are applied to the solution, which is the best of the population and is obtained from the path construction phase. The collective intelligence in the path construction phase is based on a footprint mechanism, and more footprints on the arc improve the selection chance of this arc. A selection probability is also balanced by using information about the problem (e.g., the distance between nodes for a traveling salesman problem). The performance of the proposed method has been investigated on 25 traveling salesman problems and compared with state-of-the-art algorithms. The experimental comparisons show that the proposed method produced comparable results for the problems dealt with in this study.

## 1. Introduction

The solution of optimization problems with different characteristics, such as single-objective and multi-objective discrete problems [1,2] or continuous problems [3,4], with meta-heuristic algorithms, is becoming increasingly common. Recently, swarm intelligence-based intelligent optimization algorithms have been proposed and successfully used. Swarm-based intelligent optimization algorithms are often the product of collective intelligence and populational behaviors. The ant system (AS) algorithm, which is a prominent type of swarm intelligence algorithm, was introduced by Dorigo, inspired by behaviors demonstrated by real ants between their nest and food source [5,6]. The particle swarm optimization (PSO) algorithm was investigated by Eberhart and Kennedy in 1995, who were inspired by bird flocking and fish schooling [7,8]. In 2005, Karaboga [9] introduced an artificial bee colony (ABC) algorithm that simulates the foraging and dancing behaviors of real honey bee colonies [10] for solving continuous optimization problems. In 2015, a tree-seed algorithm inspired by relationships between trees and their seeds was proposed for solving continuous optimization problems [11]. A common feature of these algorithms is information sharing about solutions among individuals in the swarm while optimization problems are solved. For instance, artificial agents in AS leave pheromones for other following ants to track, and bees in ABC perform a peculiar dance in order to share position information about good-quality solutions. Potential solutions, called particles in PSO, are affected by the global best solution of the population for producing new solutions. While seeds are produced by trees, the relation between trees is used in the production procedure. Thus, the collective intelligence in these algorithms consists of sharing information and some peculiar behaviors.

The traveling salesman problem (TSP) is a well-known NP-hard problem in the operations research field which requires exponential time due to its solution depending on the number of nodes in the graph, and it can be simply described as follows: there is one salesman who visits n cities, and their aim to find out the shortest Hamilton cycle through which they can visit all the cities once and only once and finally return to the start [12]. $x_{i,j}$ is the arc between the *i^th^* and *j^th^* node, and the mathematical model of the problem is as follows:

(1) $$obj.\left(min\right)\;\sum_{i=1}^{n}\sum_{j}^{n}c_{i,j}x_{i,j}$$

subject to

(2) $$\sum_{j=1}^{n}x_{i,j}=1,\;i=0,1,...,n-1$$

(3) $$\sum_{i=1}^{n}x_{i,j}=1,\;j=0,1,...,n-1$$

(4) $$\sum_{i=1}^{n}\sum_{j=1}^{n}x_{i,j}\leq \left|S\right|-1,\;S\subset V,\;2\leq \left|S\right|\leq n-2$$

(5) $$x_{i,j}\in \{0,1\}\;and\;\forall i,j\in E$$

where Equation (1) is the objective function, which is the minimization of the total distance for the problem, Equation (2) provides only one incoming edge to a node, Equation (3) provides only one outgoing edge to a node, Equation (4) prevents the occurrence of the subtours in the solution, and Equation (5) shows the integer variables.

The TSP is widespread in many applications, especially in engineering and operations research such as machine scheduling, cellular manufacturing, and frequency assignment problems [13]. The literature on the TSP and its variants is extensive, and the reader is referred to the surveys in [14,15,16,17,18,19,20] and to the book [21]. In order to solve TSPs, two different strategies in swarm intelligence or evolutionary computation algorithms have been used: path construction (PC) and path improvement (PI) strategies. PC-based methods, such as the greedy algorithm and Christofides algorithm, create solutions step by step. PI-based methods improve randomly generated initial solutions step by step such as k-opt, v-opt, and genetic algorithms [22,23]. While the ant system uses a PC-based strategy for finding the optimum tour of the traveling salesman problem, ABC [24] and PSO [25] try to improve solutions with a PI-based strategy. In recent years, some other swarm intelligence algorithms have also been proposed for solving traveling salesman problems. Some of these algorithms are as follows: chicken swarm optimization [26], grey wolf optimizer [27], Jaya algorithm [2], bat algorithm [28], social spider algorithm [29], sparrow search algorithm [30], earthworm optimization algorithm [31], and Komodo algorithm [32]. These studies show that traveling discrete optimization algorithms based on swarm intelligence still attract the attention of researchers.

Akhand et al. [33] proposed the discrete spider monkey optimization (DSMO) algorithm to solve the TSP in their study. They compared the results of their proposed DSMO method with the results of other methods in the literature. They stated that the experimental results show the effectiveness of the proposed DSMO method on TSP and that it is a suitable method for solving this problem. Mzili et al. [34] proposed the discrete rat swarm optimization (DRSO) algorithm for solving the TSP in their study. They compared the performance of the proposed DRSO method with the results of some meta-heuristic algorithms. As a result of the comparisons, they stated that the proposed DRSO method has a better performance. Zhang et al. [35] proposed an opposition-based ant colony optimization algorithm for solving the TSP in their study. They compared the results of their proposed method with the original ant colony (ACO). According to the results of the comparison, they stated that the ant colony optimization algorithm performed better. Gharehchopogh and Abdullahzadeh [36] proposed three new discrete crow-inspired algorithms to improve the performance of the original crow search algorithm for solving TSP. They compared the performance of the three proposed algorithms with the results of other algorithms in the literature. According to the results of the comparison, the proposed algorithms have significantly better performance. Al-Gafari et al. [37] proposed three new discrete crow-inspired algorithms to improve the performance of the basic crow search algorithm for solving TSP. They compared the performance of the three proposed algorithms with the results of other algorithms in the literature. According to the results of the comparison, the proposed algorithms have a significantly better performance. Liu et al. [26] proposed the discrete chicken swarm optimization (DCSO) algorithm for solving the TSP in their study. They compared the results of the proposed method with the results of basic ant colony optimization (ACO) and genetic algorithm (GA). According to the comparison results, they confirmed the applicability and effectiveness of their proposed method. Krishna et al. [38] proposed a spotted hyena optimizer (MH-SHO) algorithm hybridized with MapReduce for TSP. They compared the results of the proposed method with the results of the basic spotted hyena optimization (SHO), particle swarm optimization (PSO), ant colony optimization (ACO) and black hole (BH) algorithms. As a result, they concluded that the proposed method is a suitable alternative for solving the TSP. Gündüz and Aslan [2] used a nearest neighbor and random permutation approach on the Jaya algorithm. They also transformed it into a form suitable for solving discrete problems. They applied the proposed Jaya algorithm to fourteen different traveling salesman problems frequently used in the literature. The experimental results show that the proposed method is a competitive and robust solver for TSP. Zhang and Yang [39] proposed the random walk discrete cuckoo search (RW-DCS) algorithm for TSP. They compared the proposed method with state-of-the-art algorithms. They stated that the experimental results show that the proposed method is stable and superior to the compared algorithms. Almazini et al. [40] solved the TSP using the plant propagation algorithm (PPA) due to the inadequacy of traditional algorithms. However, they stated that the basic version of this method was insufficient in solution quality and proposed PPGA by making improvements such as crossover and mutation on the algorithm. They stated that PPGA has a good performance compared to its basic form. They also stated that they obtained good solutions by comparing the same method with other algorithms in the literature. Jati et al. [32] proposed the discrete Komodo algorithm (DKA) for solving the TSP. They compared the performance of the proposed DKA method with some state-of-the-art algorithms and classical algorithms. As a result of the comparison, they concluded that the proposed DKA performs better. Zheng et al. [41] proposed a hybrid genetic algorithm (RHGA) for solving TSP by hybridizing the edge-assembly crossover genetic algorithm (EAX-GA) with the Lin–Kernighan–Helsgaun (LKH) local search approach. According to the experimental results, the proposed RHGA algorithm shows a superior performance compared to the compared algorithms for TSP. Zhang and Han [30] proposed the discrete sparrow search algorithm (DSSA) for solving the TSP. In order to balance the exploration and exploitation capabilities of the proposed DSSA, they integrated various strategies such as mutation and swap operators into the algorithm. They compared the performance of the proposed method with state-of-the-art algorithms. As a result, they concluded that the proposed DSSA method is a competitive and robust method. Nayyef et al. [42] proposed the HJSPSO algorithm by hybridizing jellyfish search (JSO) and particle swarm optimization (PSO) for solving TSPs. They compared the performance of their proposed hybrid method with other algorithms, as well as their baseline, by solving 20 different TSPs. They stated that HJSPSO is a robust technique that can produce promising solutions. In the work presented by Goel et al. [43], the ACO was modified using pheromone mutations. The modified ACO was named M-ACO. They used M-ACO to solve TSP, which is a well-known NP-hard problem. They also examined it in two groups as evaporation-based ACO (E-ACO) and population-based ACO (P-ACO). When they compared all these proposed methods with the basic ACO, they said that M-ACO and P-ACO obtained better results.

Swarm intelligence-based algorithms have some disadvantages such as stagnation and running time while solving TSPs. Due to the fact that all artificial agents in AS follow the same path due to pheromone intensification and the short distance among some nodes, AS shows stagnation behavior after a while. The stagnation behavior originates from the pheromone mechanism and the evaporation of the pheromone. Generally, the pheromone evaporation is much greater than the pheromone addition by the ants to the arcs. Thus, the heuristic factor or visibility term in the transfer rule of the AS causes the selection of the shorter arcs. If the nodes of the problem are very close to each other, the AS constructs paths by using only collective intelligence. The ABC algorithm and PSO start to work with random initial solutions and use an improvement-based strategy during the iterations. The PI techniques used in these methods are quite important and it also takes a long time to achieve an optimal or near-optimal solution due to starting with random solutions and they do not use the information in the problem. For eliminating the disadvantages of ABC, PSO, and AS, a new approach is proposed in this study by considering collective intelligence having a footprint mechanism in the path construction phase and path improvement technique to solve TSPs. The new approach consists of two phases named PC and PI. In the first phase, while solution space is explored by artificial constructor agents, the collective intelligence consists of footprints left on the paths. In the second phase, the artificial improver agents select and try to improve the global best solution in the population obtained by the constructors by using the neighborhood operators. By using these strategies and information sharing between the phases, a new approach has been studied in the present work.

The rest of the paper is organized as follows: Section 2 presents the proposed method for solving TSPs, and the experimental results and comparisons on TSPs are given in Section 3. The obtained results are discussed in Section 4, and the conclusion and future works are given in Section 5.

## 2. Material and Methods

The proposed method in this paper has two types of agents. The first type of the agents is in the path construction phase, and the other type of the agents is in the path improvement phase of the algorithm. It is assumed that the number of agents in the two phases is equal, and the method also tries to achieve the optimal or near-optimal solution for the optimization problem iteratively.

### 2.1. Path Construction Phase

The path construction strategy used in the proposed method is based on the footprint mechanism and distance between nodes. Agents in this phase are called “constructors”. Initially, all the constructor agents are located at the nodes of the TSP. The selection probability of other nodes to be visited, which is the same transition rule of the ACO, is calculated as follows:

(6) $$P_{i,j}=\frac{F_{ij}^{a}\times {\left[\left(\frac{1}{D_{ij}}\right)\right]}^{b}}{\sum_{k=1}^{N}F_{ij}^{a}\times {\left[\left(\frac{1}{D_{ij}}\right)\right]}^{b}}$$

where, while artificial agent is on *i^th^* node, *P_ij_* is the selection probability of *j^th^* node, *F_ij_* is the number of footprints leaved on the arc, *D_ij_* is the distance between *i^th^* and *j^th^*, *N* is the number of unvisited nodes, and *a* and *b* are significant parameters. Equation (1) is known as the transition rule in the ant colony optimization technique [5]. At a certain time, the selection of the next node is performed by using the roulette wheel and Equation (1). After all nodes of a TSP are visited, the artificial agent comes back to the first position. When Equation (1) is analyzed, the selection mechanism for the path construction uses both the collective intelligence (number of footprints) and the information of the problem (distance between nodes). Differently from the basic ant system, the collective intelligence in the algorithm does not consist of any evaporation.

The footprint mechanism is an important factor for collective intelligence occurring. At the beginning of the search, some footprints are left on all arcs. After the constructor agents have completed self TSP tours, the number of footprints on the visited arcs is increased by 1. In other words, the artificial agents left a footprint to the arc visited. But all constructors do not leave footprints on the arcs, and agents, those solutions of these agents are better than the mean solution quality of the population, leave footprints on self-paths. Therefore, when we compare the proposed algorithm and the basic ant system, the occurrence of the collective intelligence in the algorithm is different from the basic ant system algorithm because there is no evaporation in the proposed algorithm, and the solutions better than the mean of the population leave footprint on the arcs.

### 2.2. Path Improvement Phase

**Figure 1:**
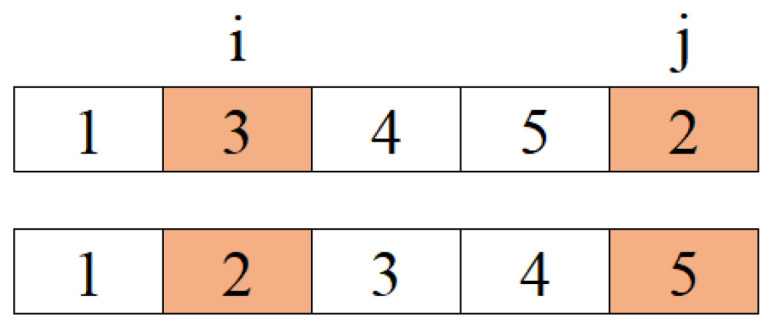
The function of RI operator.

**Figure 2:**
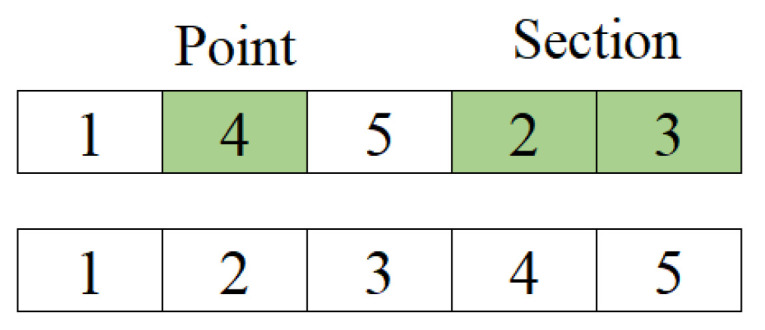
The function of RIS operator.

**Figure 3:**
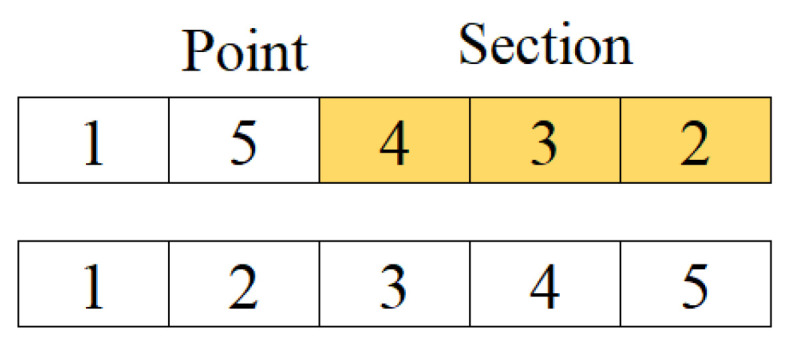
The function of RRIS operator.

The best solution obtained in the path construction phase in the iteration is compared with the best solution obtained thus far. One of them is selected and given to “improver” agents in this phase and artificial agents use neighborhood operators to improve this solution. According to Kıran et al. [44], three neighborhood operators are used for making a better tour than the best tour obtained thus far and these operators are randomly applied to the solution. If the new one obtained from the operation is better than the old one, solutions are replaced. In brief, the product of collective intelligence is used in order to obtain better solutions in this phase. The neighborhood operators used in the proposed method are random insertion of a point (RI) in Figure 1, random insertion of subsequences (RIS) in Figure 2, and reverse random insertion of subsequences (RRIS) given in Figure 3. These operators are applied to the best solution with an equal probability by each improver agent.

The RI operator adds a randomly chosen element to a randomly chosen position and shifts the rest of the sequence. If a randomly chosen point is *i* = 2 and a randomly chosen element is *j* = 5 (*i* ≠ *j*), the operation is as follows:

The RIS operator adds a subsequence randomly chosen from the subsequence to a point randomly chosen and shifts the rest of the sequence. The operation is shown as follows:

In the RRIS operator, the subsequence randomly chosen from the sequence is added to the randomly chosen point and the rest of the sequence is shifted to the right until the size of the subsequence. Before the addition, the subsequence may be inverted with a probability 50%. The function of this operator is given in Figure 3.

**Figure 4:**
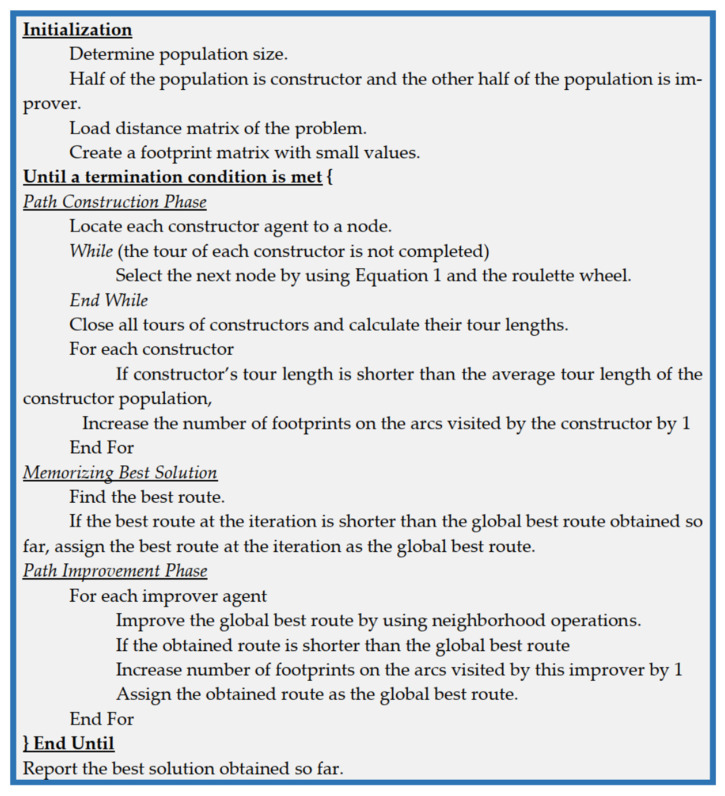
The detailed algorithmic framework of the proposed method.

Based on the aforementioned explanations, the steps of the proposed algorithm are given in Figure 4.

In the initialization of the algorithm, half of the population are constructor agents, and the rest of the population are improvers. While the algorithm is run, a potential solution is created for each constructor by using the transfer rule and the best solution obtained by the constructors have been improved in the second phase of the algorithm by the improver agents. The relation between the constructors and the improver agents is provided by the best solution in the population and footprint mechanism. The proposed algorithm can be seen like ACO, and the same transfer rule of ACO is used to create solutions in the constructor phase of the proposed algorithm. However, the evaporation mechanism is not used in our study because while the problem size is increased, the artificial agents tend to select the shortest distance among the nodes. Thus, the evaporation mechanism does not work in the transfer rule because it is too low. To overcome this issue, we used the footprint mechanism without evaporation. The second novelty is to use constructors and improvers in an algorithmic framework. As we know, all the agents in the ACO are constructors, and the best solution is only used to report at the end of algorithm.

## 3. Experimental Results

For experiments, an IBM compatible PC with a Pentium 3.4 GHz microprocessor and 2 GB of RAM was used. The algorithms we are run 30 times with random seeds for each problem, and the obtained results were reported as the best, worst, and mean. The test problems [45] used in experiments, except Oliver30 [46], and their optimum costs are displayed in Table 1, the parameters of the methods are given in Table 2, and the obtained results for these problems are given in Table 3.

In the results tables, the relative errors (RE) calculated by using the means of results are as follows:

(7) $${RE}_{k}=\frac{B_{k}-O_{k}}{O_{k}}\times 100$$

where *O_k_* is the optimum tour length of the *k^th^* problem and *B_k_* is the mean tour length obtained by 30 runs of the algorithm for the *k^th^* problem.

**Figure 5:**
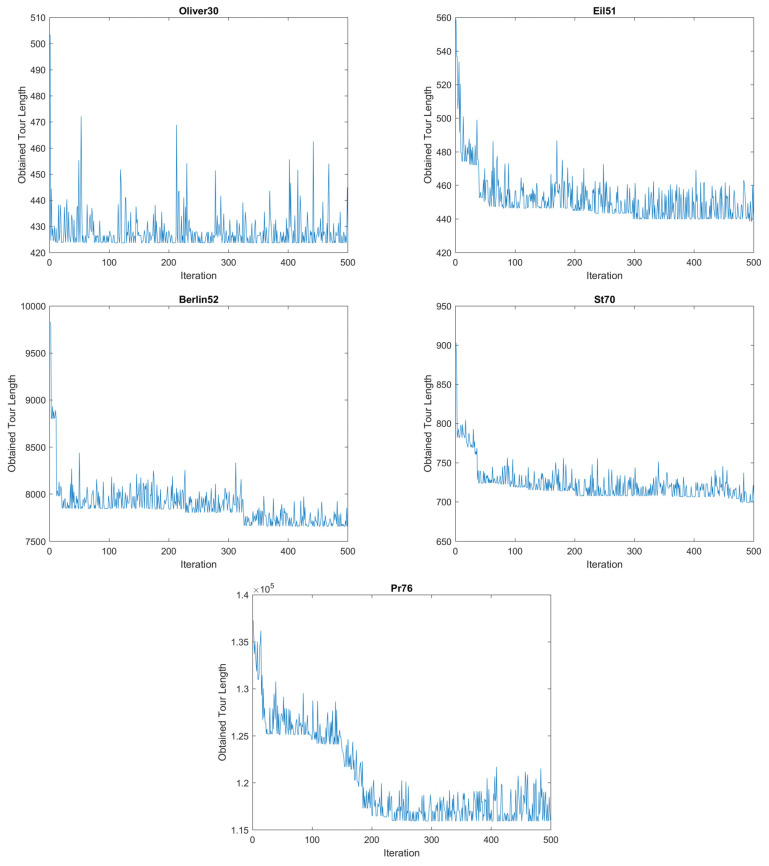
The evolution of population in the proposed approach on some test problems.

Under these conditions, the obtained results by the proposed method and ant system are reported in Table 3, and the better solutions according to RE are written in boldface font type. Based on Table 3, the proposed method is better than the AS algorithm in terms of solution quality on 20 of 25 TSPs. Because half of the population in the proposed method are improvers, all of the solutions are not re-constructed in the improvement stage and the method consumes less time than the AS algorithm. In addition, the stagnation has been prevented by the improvers and the higher-quality solution has been obtained by a new approach. Moreover, to show the search behavior of the proposed algorithm, the convergence to the global optimum of the proposed algorithm is given in Figure 5. The convergence of the algorithm to the global optimum is at an acceptable level due to using both the footprint mechanism and information about the problem. The evolution graphics of the population are shown in Figure 5 during the iterations. According to the evolution graphics on some test problems, the search space of the problem is continuously searched by the population of the proposed method effectively. In addition, the convergence graphics of the method to the optimum or near-optimum of the problems are shown in this figure.

**Figure 6:**
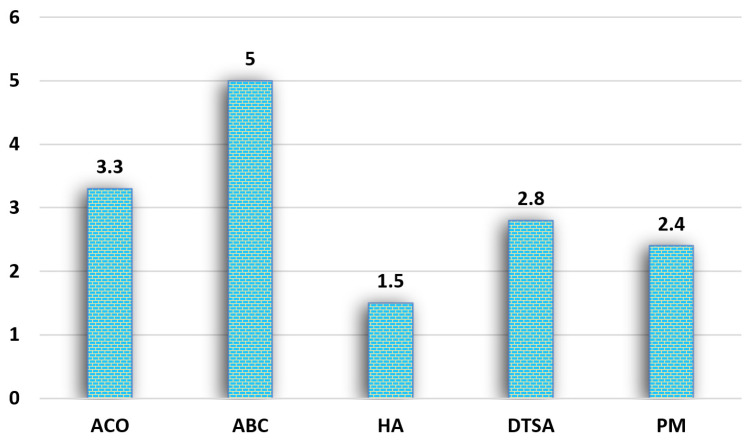
Comparison of mean ranks of the ACO, ABC, HA, DTSA, and PM in some TSP instances.

The proposed method has been also compared with the ABC, ACO, DTSA, and a hierarchic approach, briefly HA, in Table 4, in which the results of the compared algorithms (ACO, ABC, and HA) are directly taken from [47], and the results of DTSA are directly taken from [48]. The results given in Table 4 show that the hierarchic approach is better than the other algorithms in the small-sized TSP instances. The DTSA produced better results on the PR76 and Kroa100 problems than the other algorithms, and the proposed algorithm called PM in Table 4 is better than the compared algorithms in the Eil101 and Tsp225 instances. In accordance with the mean rank comparisons of the algorithms given in Figure 6, the HA is in the first rank, and the proposed algorithm is in the second rank in the comparison.

**Figure 7:**
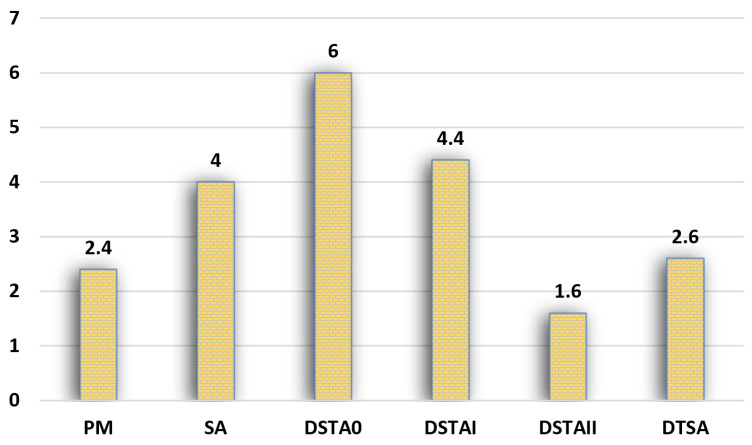
Comparison of mean ranks of the PM, SA, DTSA, and DSTAs on KRO series problems.

Another comparison has been conducted on the Kro series (KroA, KroB, KroC, KroD, KroE) TSP instances. In this experiment, the proposed method has been compared with simulated annealing (SA), DTSA, and discrete state transition algorithm (DSTA) and its variants. The results of these algorithms are directly taken from the study of [48] and the termination condition is the maximum number of function evaluations, and it was 90,000 in the referenced study. In the run of the proposed method, it was adjusted according to the number of TSPs, and because the number of nodes is 100 in Kro series problems, the number of function evaluations is calculated as 50,000. The comparison results and mean ranks are given in Table 5 and Figure 7, respectively. At the same time, Table 6 shows the comparison of the results obtained by the proposed method with the variants of ACO.

When Table 6 is analyzed, it is seen that the proposed method obtains the best average results in some TSPs according to the variants of ACO.

## 4. Results and Discussion

The performance of the proposed method for solving discrete optimization problems has been investigated on traveling salesman problems. The results obtained by the algorithm show that the method has a reasonable performance on the problems solved in this paper. The new approach was based on collective intelligence and path improvement. Some of the agents construct self-solutions, and the best of them is used in the path improvement phase. Therefore, the proposed method does not show stagnation behavior although there are more footprints on some arcs because the improvement phase in the method provides a way to discover the different tours from the best solution obtained thus far. Therefore, the agents in this phase maintain the collective intelligence in the population. The convergence characteristics of the proposed approach shown in Figure 5 are at an acceptable level since the path construction phase uses both collective intelligence and distances between nodes. Figure 5 also shows there is no stagnation in the population, and global and local searches are effective in the search process of the proposed algorithm. Additionally, a reason for obtaining good quality results was the use of information sharing over the global best solution between the path improvement and path construction phases.

## 5. Conclusions and Future Works

In this paper, a novel approach based on the ant system algorithm has been proposed and tested on the well-known traveling salesman problems. The proposed method has been compared with ant system and state-of-art algorithms on the well-known TSPs, and the proposed method has produced promising and comparable results on solving TSPs. This is based on the search mechanism included in the novel approach. The investigation into the performance of the proposed method for solving discrete optimization problems, specifically on traveling salesman problems, has yielded promising and encouraging results. The algorithm demonstrated a reasonable performance across the problems examined in this study. The novel approach, rooted in collective intelligence and path improvement, showcased a dynamic and effective strategy. The incorporation of agents constructing self-solutions, with the best among them influencing the path improvement phase, has proven instrumental in avoiding stagnation behaviors. Despite the presence of more footprints on certain arcs, the improvement phase facilitates the discovery of diverse tours, maintaining the collective intelligence within the population. Future work will focus on investigating and analyzing the performance of the proposed method on the different types of discrete optimization problems.

## Figures and Tables

**Table 1:** The TSP instances used in the experiments.

Problem	Optimum	Problem	Optimum
Oliver30	423.74	Eil51	428.87
Berlin52	7544.37	St70	677.11
Pr76	108,159.44	Eil76	545.39
Rat99	1211	Rd100	7910
KroA100	21,285.44	KroB100	22,141
KroC100	20,749	KroD100	21,294
KroE100	22,068	Bier127	118,282
Lin105	14,379	Eil101	642.31
Pr124	59,030	Pr107	44,307
Pr136	96,772	Ch130	6110
Ch150	6532.28	Pr144	58,537
KroA150	26,524	TSP225	3859
KroB150	26,130		

**Table 2:** Parameter setting of ACO and the proposed method.

Parameter	Ant System	Proposed Method
Population Size (P)	D *	D *
Maximum Cycle Number	500	500
Alpha (α)	1.0	N/A
Beta (β)	5.0	N/A
a	N/A	1.0
b	N/A	5.0
Rho (ρ)	0.65	N/A
Q	100	10

* D is the number of nodes in the traveling salesman problem.

**Table 3:** The comparison of the ant system algorithm and the proposed method on the 25 TSPs.

Problem		Ant System Algorithm					Proposed Method				
Name	Optimum	Best	Worst	Average	Std. Dev.	*RE* (%)	Best	Worst	Average	Std. Dev.	*RE* (%)
Oliver30	423.74	423.91	433.07	425.81	2.03	0.49	**423.74**	**424.46**	**423.84**	**0.20**	**0.02**
Eil51	428.87	447.37	463.42	456.52	3.83	6.45	**434.80**	**454.88**	**444.78**	**4.91**	**3.71**
Berlin52	7544.37	7663.59	7872.49	7694.30	45.53	1.99	**7544.37**	**7819.59**	**7596.93**	**70.62**	**0.70**
St70	677.11	697.02	719.27	710.54	5.91	4.94	**689.69**	**718.37**	**706.16**	**7.86**	**4.29**
Pr76	108,159.44	**114,674.63**	**118,727.64**	**116,898.74**	**977.97**	**8.08**	113,406.81	119,641.27	116,964.07	1617.86	8.14
Eil76	545.39	**559.17**	**570.36**	**565.36**	**3.35**	**3.66**	553.53	581.84	566.18	5.78	3.81
Rat99	1211.00	1269.82	1307.81	1290.49	8.29	6.56	**1242.59**	**1316.05**	**1285.80**	**18.29**	**6.18**
Rd100	7910.00	8214.34	8468.61	8359.81	60.39	5.69	**8070.45**	**8403.07**	**8217.68**	**79.46**	**3.89**
KroA100	21,285.44	22,455.89	23,271.16	22,882.11	232.25	7.50	**21,575.98**	**22,321.42**	**21,986.93**	**213.88**	**3.30**
KroB100	22,141.00	22,693.38	23,043.60	22,929.66	72.19	3.56	**22,486.55**	**23,138.72**	**22,763.13**	**171.27**	**2.81**
KroC100	20,749.00	21,218.38	21,629.82	21,479.12	109.41	3.52	**21,103.32**	**21,580.70**	**21,401.25**	**132.99**	**3.14**
KroD100	21,294.00	22,681.23	23,034.26	22,857.46	92.72	7.34	**22,057.57**	**22,784.83**	**22,408.96**	**176.76**	**5.24**
KroE100	22,068.00	22,893.72	24,020.36	23,624.95	226.28	7.06	**22,429.60**	**23,681.80**	**23,075.59**	**293.60**	**4.57**
Bier127	118,282.00	122,170.98	124,011.82	123,173.65	487.68	4.14	**120,608.54**	**124,215.06**	**122,415.63**	**974.55**	**3.49**
Eil101	642.31	674.41	704.14	693.02	7.75	7.89	**656.14**	**701.46**	**676.76**	**9.23**	**5.36**
Lin105	14,379.00	14,706.08	14,930.62	14807.13	66.35	2.98	**14,501.10**	**14,953.92**	**14,689.60**	**116.37**	**2.16**
Pr107	44,307.00	46,034.75	46,838.57	46,368.35	180.75	4.65	**44,781.22**	**46,053.97**	**45,364.00**	**301.81**	**2.39**
Pr124	59,030.00	**59,731.20**	**60,700.56**	**60,059.94**	**214.25**	**1.74**	59,553.62	60,830.49	60,316.46	322.43	2.18
Ch130	6110.00	6419.15	6579.30	6482.77	43.58	6.10	**6276.59**	**6414.22**	**6331.80**	**37.52**	**3.63**
Pr136	96,772.00	104,670.51	108,272.22	106,807.35	734.25	10.37	**102,771.33**	**108,650.39**	**105,825.77**	**1446.93**	**9.36**
Pr144	58,537.00	**58,816.80**	**58,868.72**	**58,820.60**	**10.01**	**0.48**	58,820.96	59,617.26	59,138.08	188.42	1.03
KroA150	26,524.00	27,727.74	29,006.01	28,518.77	251.62	7.52	**27,801.51**	**28,979.46**	**28,458.39**	**334.31**	**7.29**
KroB150	26,130.00	27,309.28	28,314.81	27,948.45	178.94	6.96	**27,133.53**	**28,211.93**	**27,724.68**	**256.21**	**6.10**
Ch150	6532.28	**6648.51**	**6726.27**	**6702.87**	**24.65**	**2.61**	6611.95	6788.13	6704.08	37.29	2.63
TSP225	3859.00	4112.35	4236.84	4176.08	**22.65**	8.22	**4066.95**	**4174.39**	**4130.64**	**26.49**	**7.04**

**Table 4:** The comparison of ACO, ABC, HA, DTSA, and PM in some TSP instances.

Problem	Algorithm	Best	Worst	Mean	Std. Dev.	RE	Rank
Oliver30	ACO	423.74	429.36	424 68	1.41	0.22	3
	ABC	439.49	484.83	462.55	12.47	9.16	5
	HA	423.74	423.74	423.74	0	0	1
	DTSA	N/A	N/A	428.5	4.21	1.12	4
	PM	423.74	424.46	423.84	0.2	0.02	2
Eil51	ACO	450.59	463.55	457.86	4.07	6.76	4
	ABC	563.75	619.44	590.49	15.79	37.68	5
	HA	431.74	454.97	443.39	5.25	3.39	1
	DTSA	N/A	N/A	443.93	4.04	3.51	2
	PM	434.8	454.88	444 78	4.91	3.71	3
Berlin52	ACO	7548.99	7681.75	7659.31	38.7	1.52	4
	ABC	9479.11	11,021.99	10,390.26	439.69	37.72	5
	HA	7544.37	7544.37	7544.37	0	0	1
	DTSA	N/A	N/A	7545.83	21.00	0.02	2
	PM	7544.37	7819.59	7596.93	70.62	0.7	3
St70	ACO	696.05	725.26	709.16	8.27	4.73	4
	ABC	1162.12	1339.24	1230.49	41.79	81.73	5
	HA	687.24	716.52	700.58	7.51	3.47	1
	DTSA	N/A	N/A	708.65	6.77	4.66	3
	PM	689.69	718.37	706.16	7.86	4.29	2
Eil76	ACO	554.46	568.62	561.98	3.5	3.04	2
	ABC	877.28	971.36	931.44	24.86	70.78	5
	HA	551.07	565.51	557.98	4.1	2.31	1
	DTSA	N/A	N/A	578.58	3.93	6.09	4
	PM	553.53	581.84	566.18	5.78	3.81	3
Pr76	ACO	115,166.66	118,227.41	116,321.22	885.79	7.55	3
	ABC	195,198.9	219,173.64	205,119.61	7379.16	89.65	5
	HA	113,798.56	116,353.01	115,072.29	742.9	6.39	2
	DTSA	N/A	N/A	114,930.03	1545.64	6.26	1
	PM	113,406.81	119,641.27	116,964.07	1617.86	8.14	4
KroA100	ACO	22,455.89	23,365.46	22,880.12	235.18	7.49	4
	ABC	49,519.51	57,566.05	53,840.03	2198.36	152.94	5
	HA	22,122.75	23,050.81	22,435.31	231.34	5.4	3
	DTSA	N/A	N/A	21,728.4	358.13	2.08	1
	PM	21,575.98	22,321.42	21,986.93	213.88	3.3	2
Eil101	ACO	678.04	705.65	693.42	6.8	7.96	4
	ABC	1237.31	1392.64	1315.95	35.28	104.88	5
	HA	672.71	696.04	683.39	6.56	6.39	2
	DTSA	N/A	N/A	689.91	4.47	7.41	3
	PM	656.14	701.46	676.76	9.23	5.36	1
Ch150	ACO	6648.51	6726.27	6702.87	20.73	2.61	2
	ABC	20,908.89	22,574.99	21,61748	453.71	230.93	5
	HA	6641.69	6707.86	6677.12	19.3	2.21	1
	DTSA	N/A	N/A	6748.99	32.63	3.32	4
	PM	6611.95	6788.13	6704.08	37.29	2.63	3
Tsp225	ACO	4112.35	4236.85	4176.08	28.34	8.22	3
	ABC	16,998.41	18,682.56	17,955.12	387.35	365.2792	5
	HA	4090.54	4212.08	4157.85	26.27	7.74	2
	DTSA	N/A	N/A	4230.45	58.76	9.63	4
	PM	4066.95	4174.39	4130.64	26.49	7.04	1

**Table 5:** The comparison of PM, SA, DTSA, and DSTA variants in Kro series TSP instances.

Problem	Algorithm	Mean	Std. Dev.	RE (%)	Rank
KroA100	PM	21,986.93	213.88	3.3	3
	SA	22,635	778.72	6.36	4
	DSTA0	23,213	906.11	9.07	6
	DSTAI	22,835	715.85	7.3	5
	DSTAII	21,767	221.64	2.28	2
	DTSA	21,506.78	260.55	1.06	1
KroB100	PM	22,763.13	171.27	2.81	1
	SA	23,657	445.78	6.85	4
	DSTA0	23,794	517.05	7.47	6
	DSTAI	23,734	507.38	7.19	5
	DSTAII	22,880	302.14	3.34	2
	DTSA	23,139.26	181.74	4.51	3
KroC100	PM	21,401.25	132.99	3.14	2
	SA	22,223	522.2	7.1	5
	DSTA0	22,877	709.87	10.26	6
	DSTAI	21,891	536.88	5.5	4
	DSTAII	21,378	246.34	3.03	1
	DTSA	21,817.08	217.77	5.15	3
KroD100	PM	22,408.96	176.76	5.24	2
	SA	22,911	483.01	7.59	4
	DSTA0	23,043	565.8	8.21	6
	DSTAI	22,665	592.53	6.44	3
	DSTAII	21,991	315.32	3.27	1
	DTSA	22,972.26	390.5	7.88	5
KroE100	PM	23,075.59	293.6	4.57	4
	SA	23,125	389.42	4.44	3
	DSTA0	23,738	450.82	7.21	6
	DSTAI	23,371	678.69	5.56	5
	DSTAII	22,637	166.82	2.24	2
	DTSA	22,547	121.96	1.83	1

**Table 6:** The comparison of the proposed method with ACO variants in TSP instances.

Problem	PM	M-ACO-SM [43]	M-ACO-RM [43]	E-ACO [43]	E-M-ACO [43]	E-SM-ACO [43]	P-ACO [43]	P-M-ACO [43]
Eil51	444.78	456	452	-	-	-	-	-
Berlin52	7596.93	8093	8093	8417	8091	8054	8201	8291
St70	706.16	742	734	743	726	744	774	760
Pr76	119,641	122,436	122,331	-	-	-	-	-
Eil76	566.18	566	585	-	-	-	-	-
Rat99	1285.80	1369	1369	1380	1322	1369	1389	1368
Eil101	676.76	702	754	698	703	726	748	736
Lin105	14,689.60	15,662	15,650	16,499	15,470	15,358	16,998	16,255

## Data Availability

All data are included in the main text.
